# Takayasu arteritis: differential diagnosis in a teenager with severe acute kidney injury - a case report

**DOI:** 10.1590/2175-8239-JBN-2018-0174

**Published:** 2019-01-10

**Authors:** Nara Thaisa Tenório Martins Braga, Adriana Banhos Carneiro, Kathia Liliane da Cunha Ribeiro Zuntini, Flávio Bezerra de Araújo, Elizabeth De Francesco Daher

**Affiliations:** 1 Hospital Geral de Fortaleza, Departamento de Nefrologia, Fortaleza, CE, Brasil.; 2 Hospital Infantil Albert Sabin, Departamento de Pediatria, Fortaleza, CE, Brasil.; 3 Hospital Infantil Albert Sabin, Departamento de Nefropediatria, Fortaleza, CE, Brasil.; 4 Universidade Federal do Ceará, Faculdade de Medicina, Departamento de Medicina Clínica, Fortaleza, CE, Brasil.

**Keywords:** Takayasu Arteritis, Hypertension, Acute Kidney Injury, Arterite de Takayasu, Hipertensão Arterial, Lesão Renal Aguda

## Abstract

Takayasu arteritis (TA) is a chronic granulomatous inflammatory condition of unknown cause that involves large vessels - particularly the aorta and its branches - such as the carotid, coronary, pulmonary, and renal arteries. The left subclavian artery is the most frequently involved vessel. Stenosis of the renal artery has been reported in 23-31% of the cases and may result in malignant hypertension, ischemic renal disease, decompensated heart failure, and premature death. Involvement of both renal arteries is uncommon. Early onset anuria and acute kidney injury are rare and have been reported only in a few cases in the literature. This report describes the case of a 15-year-old female with constitutional symptoms evolving for a year, combined with headache, nausea, and vomiting, in addition to frequent visits to emergency services and insufficient clinical examination. The patient worsened significantly six months after the onset of symptoms and developed acute pulmonary edema, oliguria, acute kidney injury, and difficult-to-control hypertension, at which point she was admitted for intensive care and hemodialysis. Initial ultrasound examination showed she had normal kidneys and stenosis-free renal arteries. The patient was still anuric after 30 days of hospitalization. A biopsy was performed and revealed her kidneys were normal. Computed tomography angiography scans of the abdominal aorta presented evidence of occlusion of both renal arteries. The patient met the diagnostic criteria for Takayasu arteritis and had a severe complication rarely described in the literature: stenosis of the two renal arteries during the acute stage of ischemic renal disease.

## RESUMO

A Arterite de Takayasu (AT) é uma doença inflamatória crônica, granulomatosa, de causa desconhecida, que afeta grandes vasos, principalmente a aorta e seus ramos, incluindo artérias carótidas, coronárias, pulmonares e renais, sendo a artéria subclávia esquerda o vaso mais acometido. A estenose da artéria renal é relatada em 23-31% dos casos e pode resultar em hipertensão maligna, insuficiência renal por isquemia, descompensação cardíaca e morte prematura. O acometimento bilateral de artérias renais é incomum, sendo rara a presença de anúria súbita e lesão renal aguda como sintoma inicial da doença, com poucos relatos na literatura. O caso reporta uma adolescente de 15 anos com sintomas constitucionais durante um ano de evolução, associados a problemas como cefaleia, náuseas e vômitos, com idas frequentes a serviços de emergência, sem adequada investigação clínica. Após 6 meses do início dos sintomas, a paciente evoluiu de forma grave, com quadro de edema agudo de pulmão, oligúria, lesão renal aguda e hipertensão arterial de difícil controle, sendo necessário suporte em Unidade de Terapia Intensiva e hemodiálise. A ultrassonografia inicial mostrava rins normais e artérias renais sem sinais de estenose. Após 30 dias de internamento, paciente permanecia anúrica, sendo realizada biópsia renal que se mostrou dentro dos padrões da normalidade. Angiotomografia de aorta abdominal evidenciou oclusão bilateral de artérias renais. A paciente descrita fechou critérios diagnósticos para arterite de Takayasu e manifestou uma complicação grave pouco descrita na literatura: estenose bilateral de artérias renais, ainda na fase aguda da nefropatia isquêmica.

## INTRODUCTION

Takayasu arteritis (TA) is a chronic inflammatory condition of unknown cause that involves large and medium caliber arteries, including the aorta and its main branches, and the coronary and pulmonary arteries.^1^^,^ 2 ^,^ 5 TA is seen more commonly in individuals of Asian descent, aged between 10 and 30 years, and females (80-90%). Incidence in the USA and Europe ranges from 1 to 3 cases per million population a year.^15^

Transmural granulomatous inflammation characteristically seen in TA may cause stenosis, occlusion, dilation, and/or the formation of aneurysms in the involved arteries.^4^^,^ 12 Genetic causes, infectious agents, and autoimmune factors may be connected to the progression of TA, in addition to a possible link between TA and infection by *Mycobacterium tuberculosis* (MT) as indicated in tuberculin purified protein derivative (PPD) tests.^10^^,^ 12 The insidious onset of TA often means patients are diagnosed at later stages of the disease. Progression is divided into three stages: the first revolves around systemic involvement, in which patients present with unspecific signs lasting for weeks and even months, usually left unchecked; the second stage consists of inflammation of the vessels, leading to stenosis or the formation of aneurysms; the third stage, also known as fibrotic/late-stage disease, includes manifestations resulting from limb or organ ischemia due to the narrowing or obstruction of large vessels.^7^^,^ 17

TA is diagnosed based on the criteria set out by the American College of Rheumatology, which include the following: age 40 or younger at the onset of symptoms; claudication of an extremity; decreased brachial artery pulse; > 10 mmHg difference in systolic blood pressure between arms; a bruit over the subclavian arteries or the aorta; and arteriographic evidence of narrowing of occlusion of the entire aorta, its primary branches, or large arteries in the proximal upper of lower extremities. The presence of three or more of these criteria confirm the diagnosis of TA with specificity and sensitivity above 90%.^3^^,^ 8 ^,^ 12

Treatment must be initiated with glucocorticoids 1-2 mg/kg/day for one to three months, followed by gradual decreases in dosage after 30 days combined with immunosuppressant therapy, with methotrexate as the first choice followed by azathioprine and mycophenolate mofetil; cyclophosphamide and infliximab are spared for severe and refractory cases.^4^^,^ 7

The specific effects of renal artery stenosis on the survival of patients with TA have not been described, since most studies focused on brachiocephalic involvement. However, severe hypertension is a known independent predictor of premature death and greater risk of adverse events in individuals with TA, leading to 5-year survival rates below 60%.^7^

## CASE REPORT

A 15-year-old unmarried female student and Jehovah’s witness born and residing in Fortaleza, Ceará, Brazil, arrived at our unit complaining of myalgia, asthenia, and lower back pain irradiating toward her left leg lasting for a year. Her pain had been managed with dipyrone and ibuprofen. The patient reported having intense holocranial headaches associated with nausea and vomiting for six months. She sought care several times at emergency services. Her symptoms were treated in isolation and she was diagnosed as having migraines and anxiety, which led to a prescription of fluoxetine 20 mg/day. She took the prescribed medication for six months, but her symptoms never subsided. A month prior to being hospitalized she had a dry cough and pleurisy associated with orthopnea and paroxysmal nocturnal dyspnea, which prompted her to seek care. Physical examination revealed she was tachypneic, with low oxygen saturation levels on pulse oximetry, and increased blood pressure (BP) (200/120 mmHg). Lung auscultation revealed she had diffuse bilateral crackles. She did not improve after initial measures, which included oxygen therapy, morphine, nitrate, and diuretics, and progressed to acute respiratory failure. The patient was then intubated and placed on a ventilator.

She was referred to the intensive care unit at Hospital Infantil Albert Sabin with anuria and signs of pulmonary edema. Her tests on admission showed high levels of nitrogenous wastes, and she was prescribed hemodialysis. Physical examination showed her right brachial artery pulse was decreased and a difference greater than 10 mmHg in BP between arms. The patient denied she had had previous diseases or hospitalizations. She had a family history of anxiety disorder (her mother and brother) and a cousin with pulmonary tuberculosis. After a few hemodialysis sessions with ultrafiltration, her respiratory condition improved and she was sent to a ward, although she still presented with difficult-to-control hypertension, anuria, hypercatabolism, repeated episodes of acute pulmonary edema, and indication for daily hemodialysis.

Her workup showed she had iron-deficiency anemia, increased C-reactive protein (CRP) levels, and an elevated erythrocyte sedimentation rate (ESR) (Table 1); transaminase levels and thyroid function were within normal range. Serology tests for hepatitis B, C, HIV, toxoplasmosis, cytomegalovirus, parvovirus, EBV, and syphilis were negative. A transthoracic echocardiogram revealed she had diffuse hypokinesis and a moderately dilated and dysfunctional left ventricle with an ejection fraction of 42%. No alterations were seen in the Doppler ultrasound examination of the carotid and vertebral arteries. Tests for ANA, lupus anticoagulant, anticardiolipin antibodies, anticentromere antibodies, anti-Scl 70 antibodies, antineutrophil cytoplasmic antibodies (ANCA), anti-Ro/SSA and anti-La/SSB antibodies, anti-Sm antibodies, and anti-DNA antibodies were negative; anti-β2 glycoprotein I IgM and IgG antibodies were negative; CH50, C3, and C4 were normal. Demonstration of AARB (gastric lavage) was negative. The PPD skin test read 14 mm of induration.

**Table 1:** Laboratory workup

Hemoglobin (mg/dL)	9.4	Urea (mg/dL)	120
Hematocrit (%)	29.3	Creatinine (mg/dL)	3.5
MCV (fL)	78	ESR (mm)	52
MCHC (g/dL)	32.1	CRP (mg/L)	132
Leukocytes (/mm^3^)	9200	LDH	935
Segmented (%)	63	Direct Coombs	negative
Rod (%)	0	PTH (pg/mL)	13.2
Lymphocytes (%)	29	25-hydroxyvitamin D (ng/mL)	23.2
Platelets (mil/mm^3^)	365	Total bilirubin	0.33
Iron	6	Direct bilirubin	0.05
Transferrin sat. (%)	21.3	Albumin (mg/dL)	3.9

**Figure 1:**
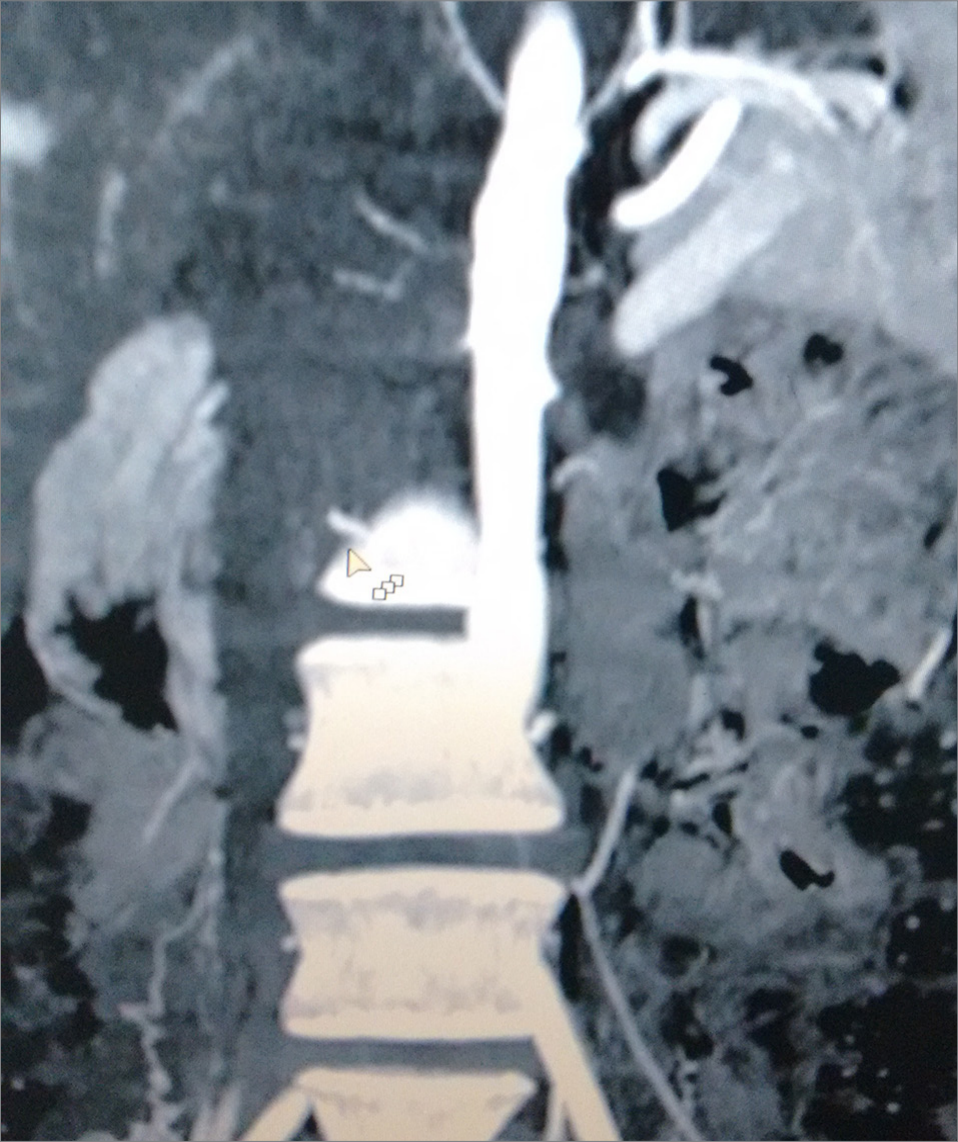
Multi-slice computed tomography angiography of the abdominal aorta showing absence of flow in the renal arteries.

Multi-slice computed tomography angiography of the chest showed nodular opacity on the upper lobe of the left lung without vascular alterations. Initial ultrasound examination of the urinary pathways showed kidneys with a normal structural pattern and location; the right kidney measured 9.3 x 4.7 x 4.1 cm with a parenchymal thickness of 14.3 mm; the left kidney measured 9.0 x 4.3 x 4.3 cm with a parenchymal thickness of 16.4 mm. Doppler flowmetry showed normal peaks of systolic and diastolic velocity and resistive index. Multi-slice computed tomography angiography of the abdominal aorta showed absence of flow in the proximal segments of the two renal arteries (Figure 1) indicating stenosis/occlusion; multiple sites with focal luminal narrowing and sites with segmental dilatation in the area where the celiac trunk emerges and in the proximal third of the superior mesenteric artery; and minor segmental luminal narrowing of the abdominal aorta in the infrarenal area 6.0 cm from the celiac trunk. Large- and medium-vessel vasculitis indicated the presence of Takayasu arteritis.

**Figure 2:**
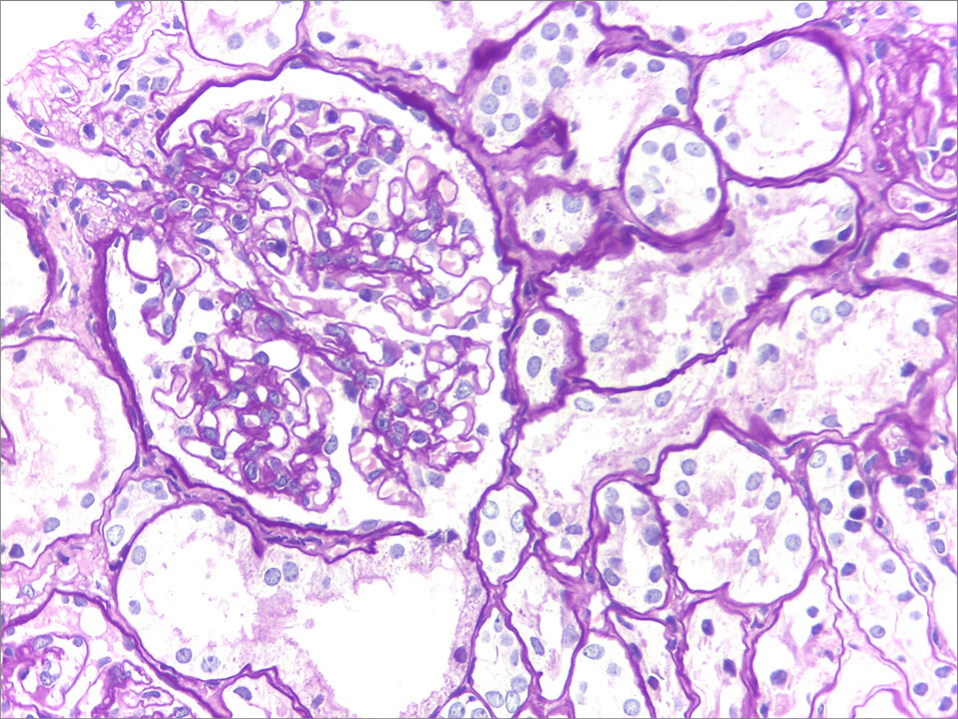
Optical microscopy image of a PAS-stained kidney biopsy specimen, 400x magnification: glomerulus with conserved cellularity and regular capillary loops.

**Figure 3:**
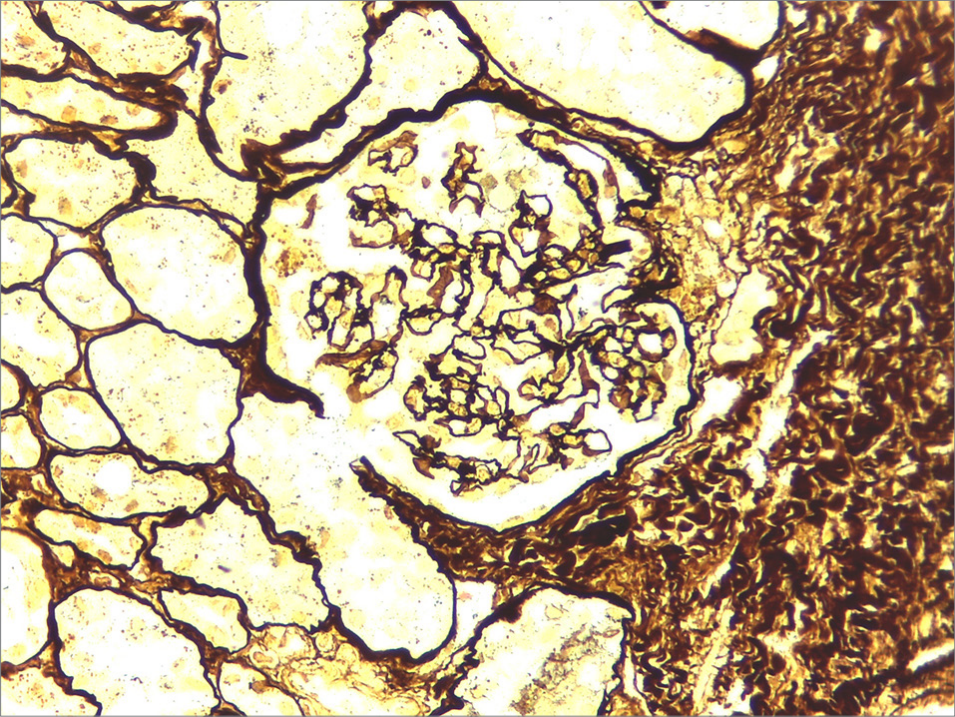
Optical microscopy image of a Jones Silver-stained kidney biopsy specimen, 400x magnification: capillary loops with regular contours.

A kidney biopsy performed on day 30 of hospitalization showed 13 glomeruli characterized as normal, global glomerulosclerosis (1/13), focal tubular atrophy with mild interstitial fibrosis, and negative immunofluorescence (Figures 2 and 3).

**Figure 4:**
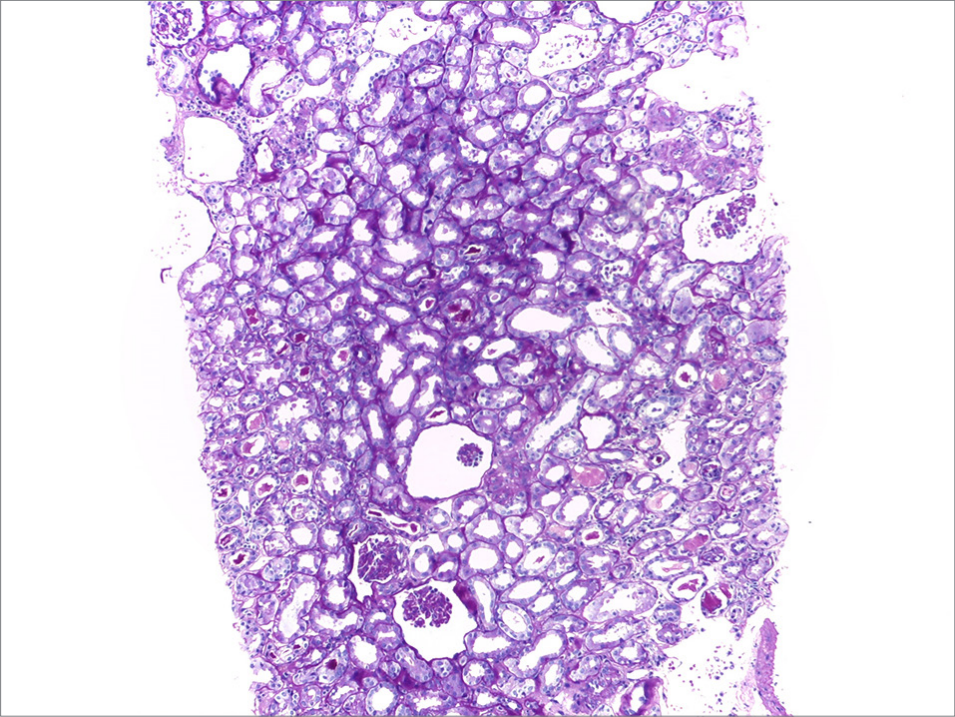
Optical microscopy image of a PAS-stained kidney biopsy specimen, 100x magnification: glomerulus with retracted tuft and relatively increased urinary space.

**Figure 5:**
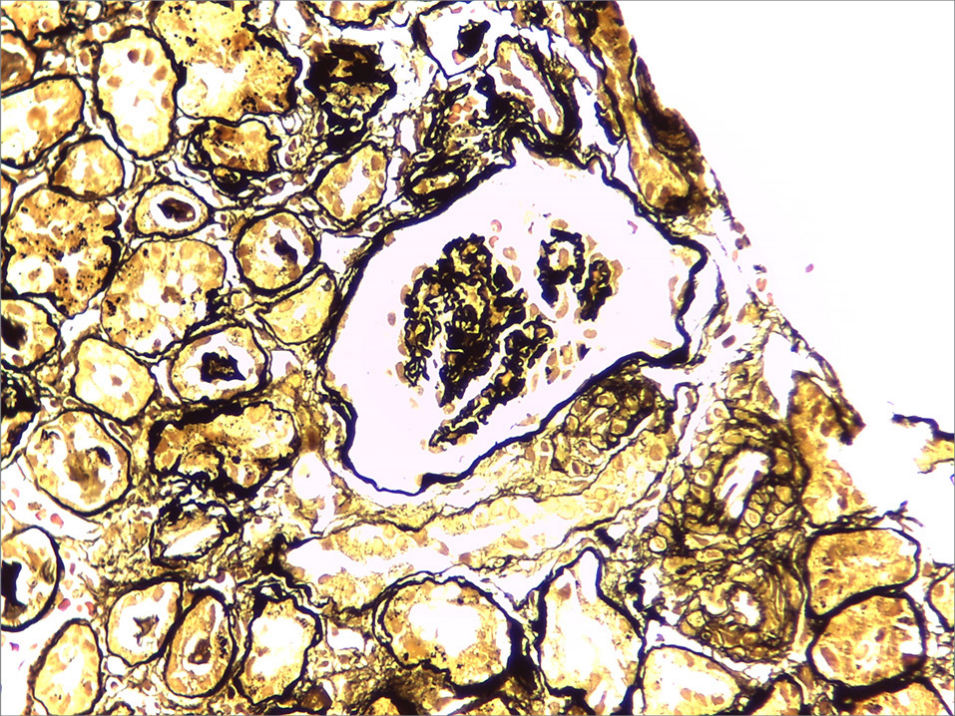
Optical microscopy image of a Jones Silver-stained kidney biopsy specimen, 400x magnification: retracted, tortuous capillary loops.

The patient was started on pulse therapy with methylprednisolone 1 g for three days followed by oral prednisone 60 mg/day. Due to the severity of her vascular involvement, she was prescribed cyclophosphamide and prophylactic isoniazid for tuberculosis. Revascularization was not indicated on account of disease activity and because she refused blood transfusions for religious reasons. Another biopsy was performed (six months after the onset of anuria) and the results showed 14 glomeruli with diffuse ischemic glomerular retraction, degenerative tubule epithelial cell alterations with signs of atrophy and moderate interstitial fibrosis, and multifocal tubulointerstitial nephritis, with negative immunofluorescence (Figures 4 and 5). The choice was made for conservative management on account of signs consistent with chronic kidney disease and the time of progression of the disease.

After a year on hemodialysis, the patient underwent a deceased-donor kidney transplant at Hospital Geral de Fortaleza. She was prescribed thymoglobulin for induction immunosuppression therapy and tacrolimus, everolimus, and prednisone for maintenance therapy. She progressed satisfactorily and was on fewer antihypertensive drugs and improved cardiac function as shown by normal electrocardiograms a year after transplantation. Her final creatinine level was 0.7 mg/dL.

## DISCUSSION

TA was described for the first time in 1908 by the Japanese ophthalmologist Mikito Takayasu, who reported the case of a 21-year-old young woman with sudden loss of eyesight, arteriovenous anastomoses around the optic disc, and no radial pulse.^2^^,^ 7 In recent years, an association has been described between TA and infection by MT. Clemente et al. described positive tuberculosis tests in 43.1% of the individuals included in a study. Our patient had a latent infection by MT evinced by a 14-mm induration on the PPD test and nodular opacity in the upper lobe of the left lung, in addition to positive epidemiology.^11^

Studies carried out in Asia found that renal artery injury associated with TA including dilatation, aneurysm, and stenosis, was reported in 30-35% of the cases. Stenosis accounted for the majority of these injuries with 23-31% of the cases.^5^ The progression of stenosis is directly linked to worsening hypertension and deterioration of renal function secondary to ischemic renal disease. Therefore, renal artery stenosis is an important prognostic factor for TA.^9^

The patient described in this report met four of the criteria for TA, had had constitutional symptoms for a year, and presented severe complications such as difficult-to-control hypertension, cardiac dysfunction, and acute kidney injury requiring dialysis, all of which indicative of renal hypertension. Renal hypertension - defined by the combination of hypertension and significant stenosis of the renal artery - is the main potentially curable cause of high blood pressure. By frequency of occurrence, the main etiologies are atherosclerosis and fibromuscular dysplasia, followed by other less common causes such as TA. Patients with severe refractory hypertension, hypertension starting before 20 or after 50 years of age, and hypertension associated with renal failure must be suspected for renal hypertension.^6^^,^ 14

Prolonged ischemia leads to gradual atrophy and loss of kidney structural integrity. The parameters around the minimum amount of stenosis required for the onset of renal ischemia have not been established, but stenosis involving more than 75% of the diameter of the renal artery has been associated with poorer outcomes. Few studies have examined the structural and functional effects of chronic decreases of perfusion pressure on renal tissue after vascular stenosis. Renal ischemia causes the release of cytokines, which by their turn trigger immune and inflammatory responses. The perpetuation of this mechanism induces renal fibrosis, with glomerular hyalinization and progressive kidney atrophy.^18^

Renovascular disease may be treated clinically, surgically, or by percutaneous procedures (with or without stent grafts). Calcium channel blockers, angiotensin-converting-enzyme inhibitors, angiotensin II type 1 receptor blockers, and beta blockers have been effectively used to treat systemic hypertension associated with renal artery stenosis. Percutaneous procedures or revascularization surgery are indicated in cases of resistant, accelerated, or malignant hypertension; progressive loss of renal function with bilateral stenosis or stenosis in individuals with one kidney; congestive heart failure or repeated episodes of acute pulmonary edema.^6^^,^ 16

Endovascular approaches such as angioplasty with or without stenting and revascularization surgery may be employed in individuals with critical stenosis, but these procedures should be carried out only after the patient has been in remission, since negative outcomes may occur in individuals with active disease.^10^^,^ 15 Recent publications on renovascular disease associated with TA have included few subjects given the low prevalence of this condition. The evidence available indicates renal angioplasty has yielded better outcomes. The immediate outcome of angioplasty is generally good, but sustaining vascular patency relies on the good management of the disease’s inflammatory activity.^15^ Treatment failure occurs primarily in cases of involvement of the aorta associated with proximal renal artery involvement.^6^

Similarly to our case, Tumeleiro et al. reported on a 25-year-old patient presented with vomiting, headaches, acute pulmonary edema, hypertension (200/140 mmHg), mildly increased creatinine level, and cardiac dysfunction. Her arteriogram revealed a 60% obstruction on the ostium of the right renal artery and severe obstruction on the ostium of the left renal artery. She had stents placed in her renal arteries (initially on the left renal artery and three years later on her right renal artery when she had an 80% obstruction) and evolved well to fewer clinical and echocardiogram signs of aortic regurgitation, normal creatinine levels, controlled BP (140/60 mmHg), and prescription of fewer antihypertensive drugs.^13^

This report described the case of a 15-year-old female with severe acute kidney injury, difficult-to-control hypertension, and cardiac dysfunction secondary to stenosis of the renal arteries by TA. Surgery was contraindicated at first for reasons of disease activity and severe anemia. After she became clinically stable, ultrasound examination of the urinary pathways and kidney biopsy revealed she had progressed to chronic kidney disease.
